# Supplementary material for: HopE and HopD Porin-Mediated Drug Influx Contributes to Intrinsic Antimicrobial Susceptibility and Inhibits Streptomycin Resistance Acquisition by Natural Transformation in Helicobacter pylori
Source: Microbiol Spectr. 2022 Mar 2;10(2):e01987-21. doi: 10.1128/spectrum.01987-21 (PMC9045298; doi:10.1128/spectrum.01987-21)
Supplement: SUPPLEMENTAL FILE 1 — Supplemental material. Download SPECTRUM01987-21_Supp_1_seq9.pdf, PDF file, 0.6 MB [file spectrum01987-21_supp_1_seq9.pdf]

Supplementary Material

HopE and HopD porins-mediated drug influx contribute to the intrinsic antimicrobial susceptibility and inhibit streptomycin resistance acquisition by natural transformation in *Helicobacter pylori*

Yixin Liu[1,2,3], Feng Yang[1,2,3], Su Wang[1], Wenjing Chi[1], Li Ding[1], Tao Liu[1], Feng Zhu[1], Danian Ji[4], Jun Zhou[4], Yi Fang[1], Jinghao Zhang[1], Ping Xiang[4], Yanmei Zhang[1,2,3*], Hu Zhao[1,2,3*]

[1] Department of Laboratory Medicine, Huadong Hospital, Fudan University, Shanghai, China

[2] Shanghai Key Laboratory of Clinical Geriatric Medicine, Shanghai, China

[3] Research Center on Aging and Medicine, Fudan University, Shanghai, China

[4] Department of Endoscopy, Huadong Hospital, Fudan University, Shanghai, China

[*]Correspondence:

Hu Zhao: ZH13701618011@163.com; Yanmei Zhang: 15618653286@163.com

Supplementary Figure S1

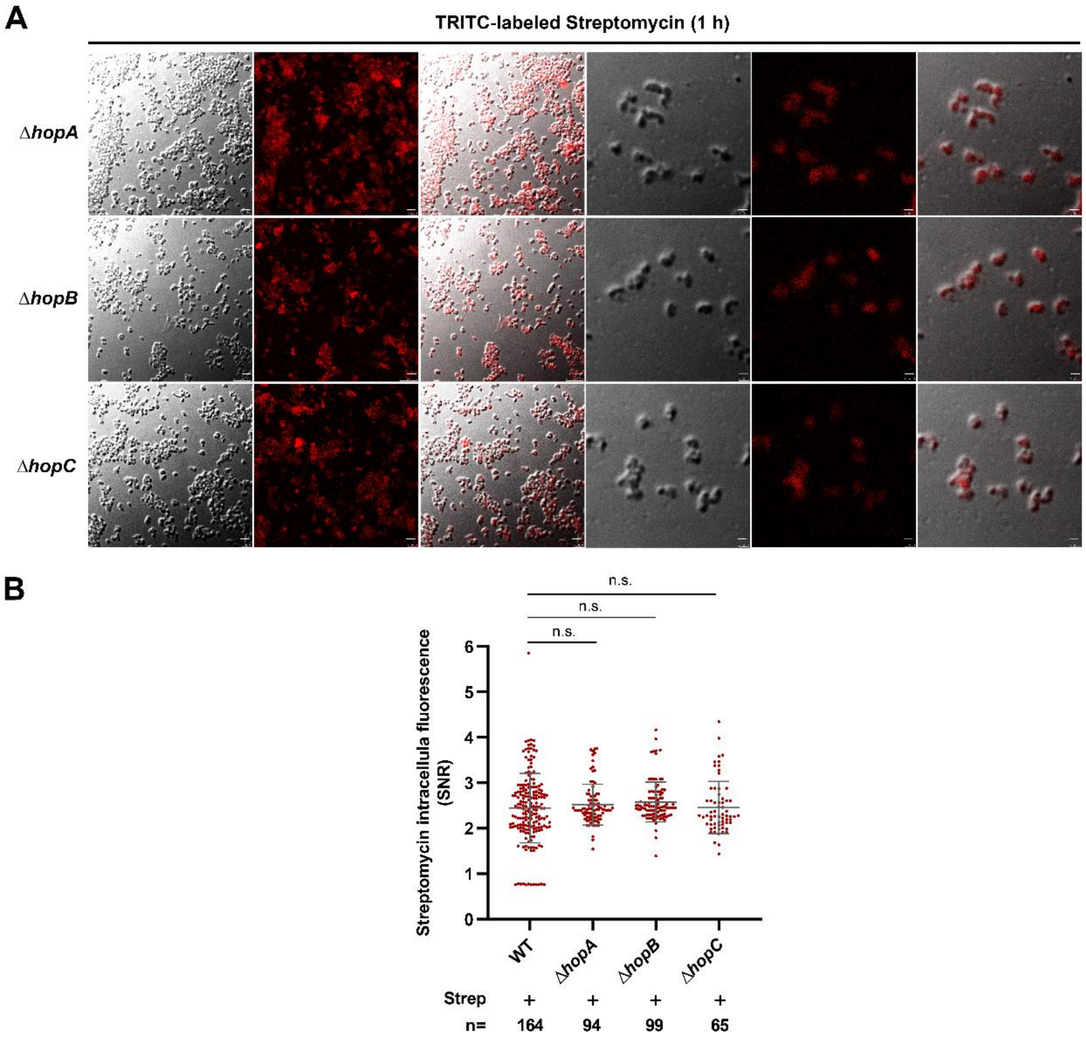

Fig. S1 HopA, HopB and HopC were not involved in streptomycin influx. (A) Representatives of the red fluorescence detection after 1 h incubation in the presence of TRITC labeled-streptomycin. An automatic red fluorescence channel and merged images of TRITC with DIC are shown. Scale bars correspond to 2.5 µm and 1 µm, respectively. (B) Quantification of streptomycin uptake by WT (using the data shown in the manuscript), Δ*hopA*, Δ*hopB* and Δ*hopC* strains. Scatter dot plots showing the single-cell quantification of intracellular fluorescence (SNR) in the population of cells after one hour of incubation with streptomycin (5 µg/ml). Same representation as in Fig. 2B. The number of cells analyzed (n) is also indicated. n.s. —not significant. Source data is provided as a Source Data file.

Supplementary Figure S2

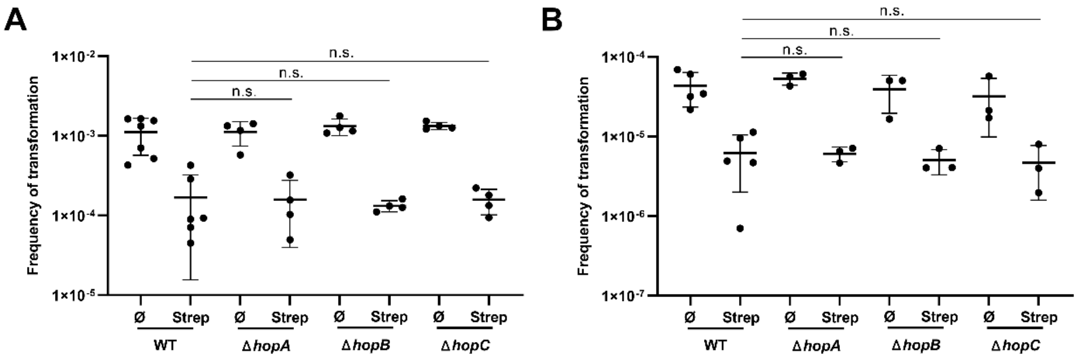

Fig. S2 HopA, HopB and HopC were not involved in streptomycin resistance acquisition by NT. Frequency of natural transformation of StrepR total chromosomal DNA (A) and rpsLmut-sfgfp linear plasmid (B) in WT (using the data shown in the manuscript), Δ*hopA*, Δ*hopB* and Δ*hopC* strains, estimated by plating assay 8 h after mixing the DNA with recipient cells, in the absence (Ø) and in the presence of streptomycin (5 µg/ml), with the mean and standard deviation calculated from at least 3 biological repeats (black dots). Source data is provided as a Source Data file.

Supplementary Figure S3

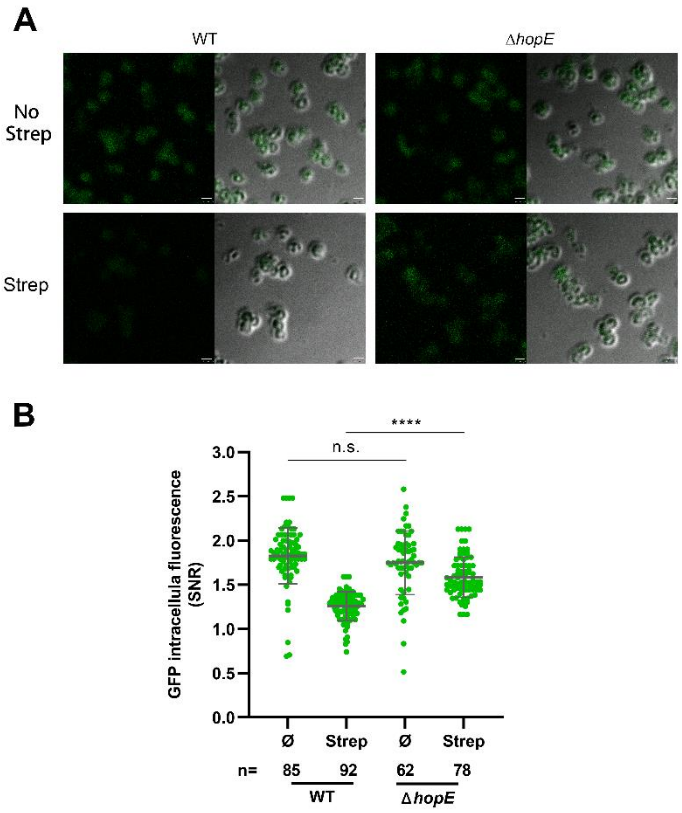

Fig. S3 Detection of sfGFP production after transformation of p946-*P*$_{ureA}$*sfgfp* plasmid in the absence and presence of streptomycin in WT and Δ*hopE* strains. We constructed strains expressing sfGFP. The chemically synthetic dsDNA containing 600 bp around HpUreA start codon and 711 bp fragment gene coding for sfGFP was cloned into pUC19/SmaI vector to generate the pUC19-*P*$_{ureA}$*sfgfp* plasmid. The plasmid was purified, linearized and transformed as described in the manuscript. (A) Representatives of microscope sfGFP detection 3 h after incubation in the absence and presence of streptomycin. Automatic GFP channel and merged images of GFP and DIC are shown. Scale bars correspond to 1.5 µm. (B) Scatter plots showing the single-cell quantification of intracellular fluorescence of sfGFP (SNR) at 3 h after incubation of pUC19-*P*$_{ureA}$*sfgfp* plasmid with bacteria, in the absence and presence of streptomycin (5 µg/ml). Same representation as in Fig. 4F. The number of cells analyzed (n) for one representative experiment is shown. Source data is provided as a Source data file.

Supplementary Figure S4

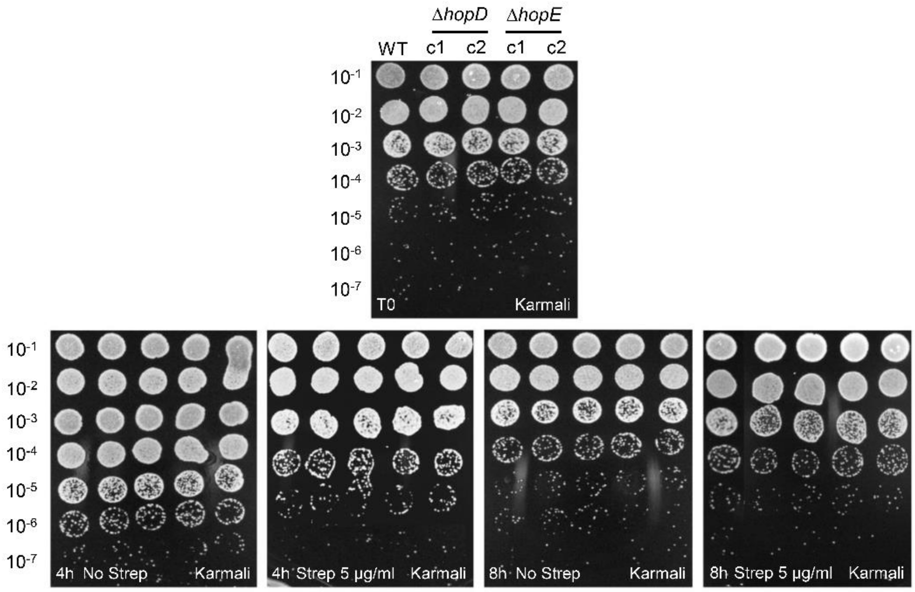

Fig. S4 Viability of WT, Δ*hopD* and Δ*hopE* strains after transient exposure to streptomycin. Platting assay showing that transient (4 and 8 h) exposure to streptomycin 5 µg/ml has no significant effect on the viability of WT, Δ*hopD* and Δ*hopE* strains (c1 and c2 denote that the results are shown for two independent clones).

Supplementary Table S1

Table S1 Sub-inhibitory concentrations for Δ*hopE* and/or Δ*hopD* mutants of the antibiotics used in Fig. 1.

**Antibiotics**	**Sub-inhibitory concentrations (µg/ml)**
metronidazole	1
clarithromycin	0.05
levofloxacin	0.01
amoxicillin	0.1
tetracycline	0.1
rifampin	0.1
streptomycin	1
